# Genetic diversity and occurrence of permethrin resistance-associated mutations in a population of head lice (*Pediculus humanus capitis*) from Seville, Spain

**DOI:** 10.1093/jisesa/ieag110

**Published:** 2026-10-01

**Authors:** Julia Rivero, Antonio Zurita, Cristina Cutillas, Angela M García-Sánchez, Rocío Callejón

**Affiliations:** Department of Microbiology and Parasitology, Faculty of Pharmacy, University of Seville, Seville, Spain; Department of Microbiology and Parasitology, Faculty of Pharmacy, University of Seville, Seville, Spain; Department of Microbiology and Parasitology, Faculty of Pharmacy, University of Seville, Seville, Spain; Department of Microbiology and Parasitology, Faculty of Pharmacy, University of Seville, Seville, Spain; Department of Microbiology and Parasitology, Faculty of Pharmacy, University of Seville, Seville, Spain

**Keywords:** head lice, *Pediculus humanus capitis*, insecticide resistance, permethrin

## Abstract

Human head lice (*Pediculus humanus capitis* De Geer, 1767) remain a public health concern, largely due to their widespread resistance to commonly used pediculicides. This study assessed genetic diversity and permethrin resistance in head lice collected from individuals in Seville, Spain. A total of 402 lice were sampled, of which 27 specimens were selected for detailed morphological, molecular, and phylogenetic analyses. Molecular characterization based on mitochondrial markers (*cox*1, *co*b and 12S) revealed a specific pattern of genetic diversity, identifying two distinct clades (A and B), with low sequence similarity between them, and clade B predominating in the sampled population. Geometric morphometric analyses revealed significant global size differences between clades A and B, with clade B lice being the largest specimens. All analyzed lice carried three mutations (M815I, T917I and L920F) in the voltage-sensitive sodium channel (VSSC) gene, indicating fixation of alleles associated with permethrin resistance. These findings provide preliminary evidence of permethrin resistance-associated mutations in head lice from Seville and underscore the need for larger studies to determine their frequency and epidemiological significance. Moreover, this study represents the first molecular characterization of head lice in this region and supports the combined use of morphometric and molecular approaches to better understand lice diversity and resistance patterns.

## Introduction

*Pediculus humanus*, commonly known as the human louse, includes two ecotypes of a single species and has long posed a significant public health challenge: the body louse (*P. humanus humanus*), which resides in clothing, primarily infests individuals in conditions of poverty and inadequate hygiene, whereas the head louse (*P. humanus capitis*) lives on the scalp and is found worldwide, independent of hygiene levels (Light et al. 2008a, Weisbroth 2024). Both ecotypes are obligate hematophagous ectoparasites that have maintained a close evolutionary association with humans over millions of years (Reed et al. 2004).

Human infestation with these lice causes pediculosis, characterized by symptoms such as intense itching (pruritus), skin rashes (urticaria) and, rarely, anemia (Ouarti et al. 2023). Various treatment options are available for pediculosis capitis, including insecticidal products (eg permethrin and ivermectin), physically acting products (eg dimethicone), and mechanical removal by wet combing. Current treatment recommendations vary between countries and should take into account local patterns of insecticide resistance (Meister and Ochsendorf 2016, Leung et al. 2022).

Permethrin resistance, referred to as knockdown resistance (kdr), is caused by three-point mutations in the gene encoding the α-subunit of the sodium channel, which is responsible for the depolarization of nerve cells (Dong 2007). This resistance leads to amino acid substitutions M815I, T917I, and L920F in the paraorthologous voltage-sensitive sodium channel (VSSC) α-subunit (Lee et al. 2003). Several authors have reported that these mutations are commonly found together as a resistant haplotype (Clark 2010). A recent meta-analysis estimated the global prevalence of pyrethroid resistance in head lice to be 59%, with permethrin resistance reaching 65% (Abbasi et al. 2023). High resistance frequencies have been reported in numerous countries, particularly in regions where permethrin has been extensively used for several decades.

Similarly, various phylogenetic studies of human lice have been conducted using mitochondrial DNA, primarily focusing on the 12S, *co*b, and *cox*1 genes. These studies have revealed that *P. humanus* is divided into six subclades, or haplogroups, each with a distinct geographical distribution (A–F). Head lice, in contrast to body lice that are confined to clades A and D, include the full range of genetically diverse clades (Ashfaq et al. 2015, Amanzougaghene et al. 2016, 2017, 2019, Hammoud et al. 2021). Despite the diversification of clades and their geographical distribution, the ecotypes of *P. humanus* are morphologically and biologically nearly identical (Light et al. 2008b).

Understanding the genetic diversity and insecticide resistance of *P. humanus* is crucial for improving the management of pediculosis worldwide. Molecular characterization of louse populations provides valuable insights into population structure, patterns of spread, and the emergence of resistance, thereby supporting surveillance programs and the development of effective treatment recommendations.

The aim of this study was designed to characterize the genetic diversity of head lice populations in Seville, Spain, and to assess the prevalence of permethrin-resistance-associated mutations.

## Materials and Methods

### Sample Collection

Between October and November 2023, a total of 402 head lice were collected from 22 individuals attending specialized lice removal centers in Seville, Spain. The study population comprised 19 females and 3 males, ranging in age from children to adults, with children representing the majority of the participants.

The study protocol was approved by the *Comité de Ética en la Investigación de la Universidad de Sevilla* (SICEIA ID: 2026-0800). Written informed consent was obtained from all adult participants or from the parents or legal guardians of minors prior to sample collection. Epidemiological information, including age, hair color, hair washing frequency, and previous use of pediculicidal products, was recorded using a standardized questionnaire. The number of lice collected varied among the examined individuals. In addition to the collected adult lice, nymphs at various developmental stages were also identified. The head lice collected were individually stored in Eppendorf tubes, one for each louse, without preservatives, and frozen at −20 °C until further analysis.

For the analyses, lice were collected from each host with the intention of sampling one male and one female per individual. However, this was not always feasible, as some hosts harbored only a single louse or specimens of the same sex, resulting in a total of 27 lice (14 males and 13 females). First, morphological and biometric analyses of the specimens were performed, followed by molecular studies.

### Morphological and Morphometric Analysis

For morphological analysis, whole specimens were examined under an optical microscope for specific classification and mounted on glass slides with saline solution. The mounted lice were examined again for a more detailed morphological analysis using a BX61 microscope (Olympus, Tokyo, Japan) and submitted to image capture processes with the imaging software cellSens Standard version 4.2 (Olympus, Tokyo, Japan). The measurement images of each louse were made using the image analysis software Image-PRO v11 (Media Cybernetics, Rockville, MD, USA). Descriptive univariate statistics (arithmetic means, SDs, and coefficient of variation) for all parameters were determined. The data were subjected to one-way ANOVA (analysis of variance) for statistical analysis of the parameters. The results were statistically significant when *P* < 0.05. Statistical analysis was performed using Microsoft Excel 365. In addition, biometric characteristics of lice were compared, and the most significant parameters were assayed for a morphometric study.

Morphometric data were explored using multivariate analysis in three measurements (Global Area, Tibia width and Tarsal claw length) by principal component analysis (PCA), comprising a technique for summarizing most of the variation in a multivariate dataset in a few dimensions (Rohlf and Marcus 1993, Dujardin and Le Pont 2004). The analyses were carried out using BAC v.2 (Dujardin 2002).

Regarding the measurements of louse legs, we selected the first pair of legs, specifically the right foreleg, because its articulations were clearly identifiable and it was generally better positioned in mounted specimens for photography, facilitating image standardization for geometric morphometric analysis.

### DNA Extraction

For the molecular analysis, prior to DNA extraction, each louse was thawed and individually homogenized using a TissueLyser III (Qiagen, Hilden, Germany) to ensure complete tissue disruption. Total genomic DNA was isolated using the DNeasy Blood and Tissue Kit (Qiagen) in accordance with the manufacturer’s guidelines. The quality of the extracted DNA was evaluated using 0.8% agarose gel electrophoresis stained with SYBR Safe DNA gel stain.

### Identification of Louse by PCR Assays and Sequencing

The molecular markers used were *cox*1, *co*b, and 12S mtDNA. These were amplified through polymerase chain reaction (PCR) using a thermal cycler (Eppendorf AG, Hamburg, Germany). The final reaction volume used for the PCR analysis was 50 µl, which included 25 µl of GoTaq G2 Green Master Mix (Promega, Madison, WI, USA), 5 µl of each primer (10 µM), 5 µl of template DNA, and nuclease-free water to 50 µl. Detailed information on the primers utilized can be found in Table 1. For *cox*1, *co*b and 12S, molecular markers, the thermal cycling profile included an initial incubation step at 94 °C, 35 cycles of 1 min each at 94 °C, 30 s at 55–56 °C and 1 min at 72 °C, followed by a final extension step of 5 min at 72 °C (Raoult et al. 2008, Amanzougaghene et al. 2019).

**Table 1.** Primers used for amplifying DNA regions

Molecular marker	Primers	Sequence (5′ to 3′)	Base pair sizes	Reference
***cox*1**	cox1F	5′GGAGTGAGTTCGATTTTAG3′	286	Amanzougaghene et al. 2019
	cox1R	5′GTGCTGAGGAAAGAAAGTC3′		
***Co*b**	cytbF1	5′GAGCGACTGTAATTACTAATC3′	272	Raoult et al. 2008
	cytbR1	5′CAACAAAATTATCCGGGTCC3′		
**12S**	12SF	5′CAGCACTAGCGGTCATACAT3′	566	Amanzougaghene et al. 2019
	12SR	5′AATGACGGGCGATATGTAC3′		
**VSSC α-subunit gene**	5HL-QS	5′ATTTTGCGTTTGGGACTGCTGTT3′	752	Kwon et al. 2008
	3HL-QS	5′CCATCTGGGAAGTTCTTTATCCA3′		

PCR products were analyzed on 2% agarose gels prepared with Tris-Borate-EDTA (TBE) buffer and stained with SYBR Safe. Purification of the amplified products was conducted using the Wizard SV Gel and PCR Clean-Up System Kit (Promega, Madison, WI, USA). The purified DNA was concentrated and then sequenced by Stab Vida (Lisbon, Portugal).

### Molecular and Phylogenetic Analysis

To assemble and edit the sequences, the forward and reverse reads were analyzed using Multiple Sequence Alignment by CLUSTALW, generating a consensus sequence for each specimen. The resulting sequences were compared to those available in the National Center for Biotechnology Information (NCBI) GenBank database using the BLASTn algorithm for species identification (https://blast.ncbi.nlm.nih.gov/Blast.cgi). All obtained sequences were subsequently submitted to the NCBI GenBank database.

To assess clade diversity in human lice, the head lice sequences obtained for all molecular markers in this study (*cox*1, *co*b, and 12S) were combined and compared with globally reported *P. humanus* sequences from previous studies obtained from the GenBank database (Supplementary file 1: Table S1). Moreover, to analyze the similarity between all *P. humanus* marker sequences obtained in this study and those of other *Pediculus* species, nucleotide differences per sequence were analyzed using the Compute Pairwise Distances function with the number of differences method in MEGA X v10.1.8 (Kumar et al. 2018).

Phylogenetic trees were inferred using nucleotide data through two methods: Maximum Likelihood (ML) and Bayesian Inference (BI). ML trees were generated using the PHYML package (Guindon and Gascuel 2003), while BI trees were produced with MrBayes v.3.2.6 (Ronquist and Huelsenbeck 2003). The best-fit substitution model for the dataset was determined using jModelTest (Posada 2008) according to the Akaike Information Criterion (AIC) (Huelsenbeck and Rannala 1997, Posada and Buckley 2004).

For analyses involving the concatenation of three mitochondrial markers (*cox*1, *co*b, and 12S), the sequence accession numbers used for phylogenetic tree reconstruction are listed in Supplementary file 1: Table S1. BI analyses were partitioned by gene, with models for individual genes selected using jModelTest. ML inferences employed the GTR + G + I model (general time-reversible model with gamma-distributed rate variation and a proportion of invariable sites) for all markers. Topology support was assessed using bootstrapping (heuristic option) with 1000 replicates (Felsenstein 1985).

All markers, using MrBayes (v.3.2.6), BI analyses used nst = 6 with invgamma rates. The standard deviation of split frequencies was monitored to confirm convergence, with chains sampled every 500 generations over a total of 10 million generations. The first million generations were discarded as burn-in, based on log-likelihood evaluations. Bayesian posterior probabilities (BPP) were calculated and converted to percentages for clade support.

### Resistance Analysis

To analyze permethrin resistance in lice, the partial gene of the VSSC α-subunit was used. Detailed information on the primers utilized can be found in Table 1. To amplify this partial gene, the thermal cycling profile comprised an incubation step at 95 °C, 35 cycles of 30 s each at 95 °C, 30 s at 63 °C, and 1:30 min at 68 °C, followed by a final extension step of 5 min at 68 °C (Kwon et al. 2008).

PCR products were analyzed on 2% agarose gels prepared with Tris-Borate-EDTA (TBE) buffer and stained with SYBR Safe. The amplification products were purified using the Wizard SV Gel and PCR Clean-Up System Kit (Promega, Madison, WI, USA). Subsequently, the same procedure for sequencing and analysis previously described for the mitochondrial markers was followed until the sequences were obtained and compared with those available in the GenBank databases.

## Results

### Morphometric Analysis

Statistical tests showed several significant measurements for subsequent morphometric analyses. Therefore, the following parameters were used: Global Area, Tibia width, and Tarsal claw length (Table 2; Fig. 1). The Global Area was defined as the Area of the whole arthropod (head, thorax, and abdomen), excluding the legs.

**Fig. 1.**
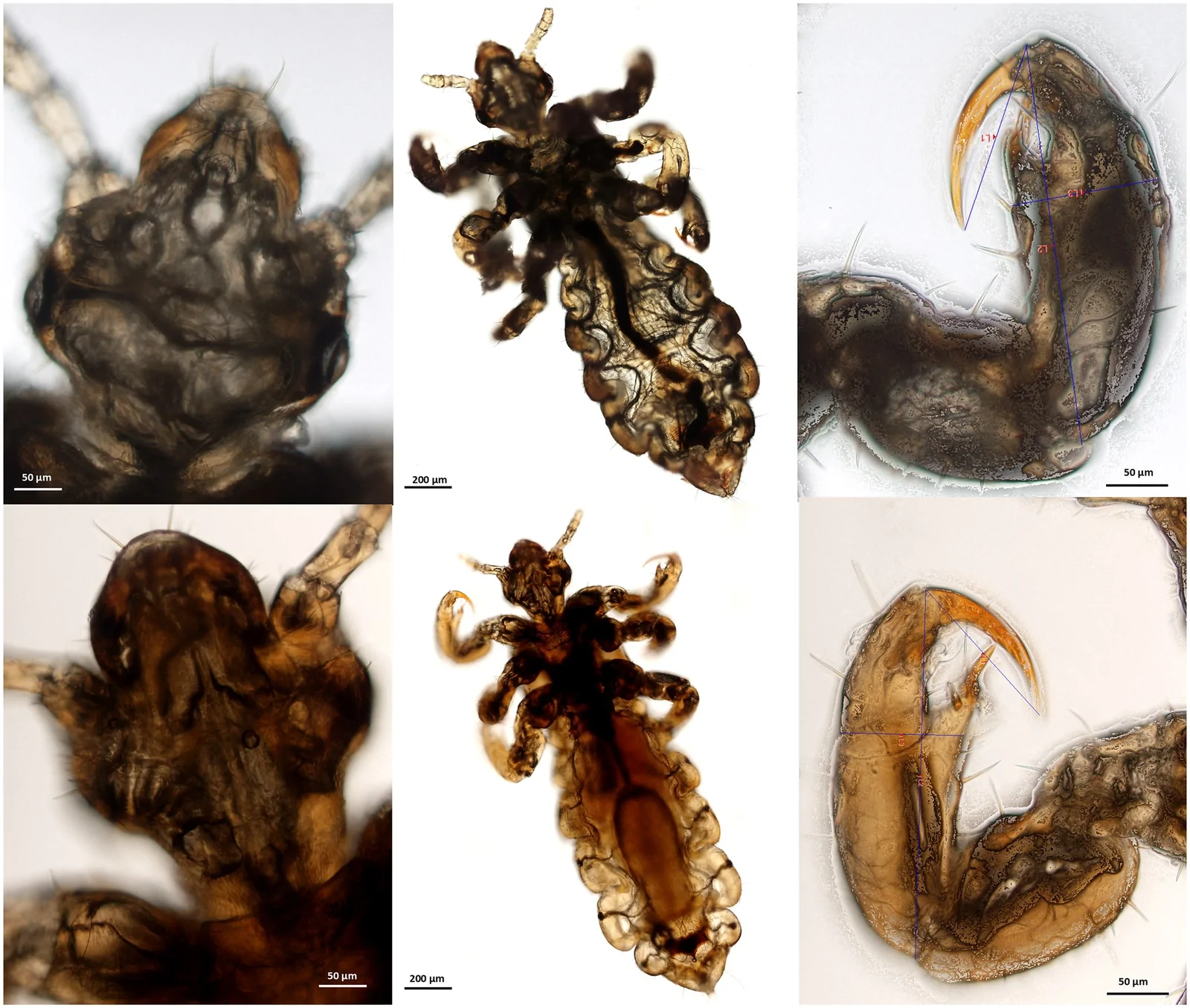
Morphological comparison of *P. humanus* belonging to clades A (top row) and B (bottom row). From left to right, panels show the head, the complete arthropod in dorsal view, and the leg featuring the tarsal claw. Scale bars are indicated in each panel.

**Table 2.** Biometrical data of males and females of clades A and B from *P. humanus* collected from Seville (Spain)

	Male									
	Clade A					Clade B				
Measured structures	MAX	MIN	Mean	SD	VC	MAX	MIN	Mean	SD	VC
**Global measures**										
**Area (µm^2^)** ^a^	883883	641620	765228	98960	13	1032332	735902	874207	118214	14
**Roundness** ^a^	2.43	2.21	2.33	0.11	5	2.33	2.15	2.21	0.05	2
**Circularity**	0.28	0,25	0,27	0.02	6	0.29	0.26	0.27	0.01	3
**Perimeter (µm)** ^a^	4957	4428	4717	225	5	5308	4478	4919	325	7
**Length (µm)** ^a^	1994	1796	1911	90	5	2169	1841	2009	125	6
**Width (µm)** ^a^	534	423	461	50	11	578	400	481	57	12
**Head measures**										
**Area (µm^2^)** ^a^	98375	77228	86373	9000	10	94111	63747	80662	8535	11
**Roundness** ^a^	1.33	1.29	1.31	0.02	2	1.40	1.10	1.26	0.09	7
**Circularity** ^a^	0.67	0.60	0.63	0.03	5	0.86	0.64	0.74	0.08	11
**Perimeter (µm)** ^a^	1273	1137	1189	60	5	1229	937	1126	81	7
**Length (μm)** ^a^	406	377	388	13	3	391	294	352	34	10
**Width (μm)** ^a^	308	283	294	12	4	317	252	283	18	6
**Thorax length (μm)**	504	469	485	14	3	600	385	515	67	13
**Abdomen length (μm)** ^a^	1106	944	1036	78	8	1273	970	1134	96	8
**Legs measures**										
**Tibia length (µm)**	380	302	338	32	10	393	303	356	29	8
**Tibia width (µm)** ^a^	136	106	123	13	10	156	122	142	11	8
**Tarsal claw length (µm)** ^a^	204	166	184	21	11	206	165	191	13	7

	Female									
	Clade A					Clade B				
Measured structures	MAX	MIN	Mean	SD	VC	MAX	MIN	Mean	SD	VC
**Global measures**										
**Area (µm^2^)** ^a^	1588139	971394	1158574	245693	21	2429893	1285064	1714855	366129	21
**Roundness** ^a^	2.52	2.21	2.33	0.12	5	2.72	2.47	2.55	0.08	3
**Circularity**	0.30	0.26	0.28	0.02	6	0.30	0.27	0.28	0.01	4
**Perimeter (µm)** ^a^	6641	5535	5790	478	8	8850	6340	7383	838	11
**Length (µm)** ^a^	2610	2161	2284	185	8	3236	2448	2771	273	10
**Width (µm)** ^a^	641	374	493	99	20	626	532	573	38	7
**Head measures**										
**Area (µm^2^)** ^a^	104863	80307	92498	10500	11	119415	89757	104446	10812	10
**Roundness** ^a^	1.23	1.09	1.17	0.06	5	1.30	1.21	1.25	0.03	3
**Circularity** ^a^	0.83	0.68	0.75	0.05	7	0.79	0.65	0.71	0.05	7
**Perimeter (µm)** ^a^	1255	1049	1166	78	7	1379	1175	1277	68	5
**Length (μm)** ^a^	421	336	380	38	10	472	353	417	43	10
**Width (μm)** ^a^	341	266	311	30	10	352	316	334	14	4
**Thorax length (μm)**	555	443	500	46	9	592	361	524	75	14
**Abdomen length (μm)** ^a^	1682	1231	1373	180	13	2226	1540	1819	277	15
**Legs measures**										
**Tibia length (µm)**	400	213	332	74	22	401	231	322	51	16
**Tibia width (µm)** ^a^	130	103	117	10	9	133	101	114	11	10
**Tarsal claw length (µm)** ^a^	175	151	160	10	6	185	138	162	20	12

MAX = maximum, Mean = arithmetic mean, MIN = minimum, SD = standard deviation, VC = coefficient of variation (percentage converted).

a Significant differences between both groups of males (*P* < 0.05). Roundness is calculated as: (perimeter of the head^2^)/(4 × pi × Area of the head). Circularity is calculated as the ratio of the area of the louse to a circle whose diameter is equal to the louse’s maximum feret.

After morphological and molecular identification, two distinct groups were obtained: 9 specimens from clade A (four males and five females) and 18 from clade B (ten males and eight females) were measured according to 17 different parameters (Table 2). Moreover, only one host presented a mixed infestation, with two lice collected from the same individual: one female belonging to clade A and one male belonging to clade B. In all other hosts, all collected lice belonged to the same mitochondrial clade, either A or B. Overall, morphometric data revealed differences between specimens from both clades, as global and specific body measurements, such as area and abdomen length, tended to show higher values in clade B individuals, as evidenced by the mean values reported in Table 2. Furthermore, these results were compared with those obtained by PCA, consisting of the regression of each character separately on the within-group first principal component (PC1), which is a multivariate estimate of size. Thus, the PCA carried out with adult male and female *P. humanus* variables from clade A and clade B was significantly correlated with PC1, contributing 86% to the overall variation. The resulting maps (Fig. 2) clearly illustrate global size differences in the populations analyzed, including a bigger size in female *P. humanus* from clade B.

**Fig. 2.**
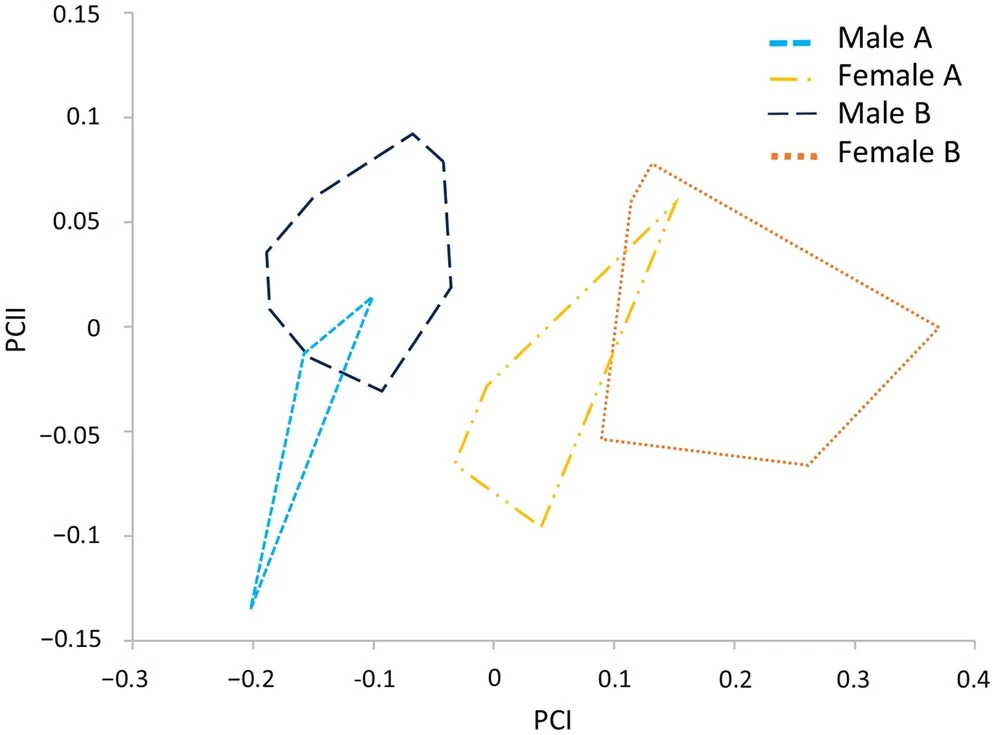
Factor map corresponding to *P. humanus* male and female adults from clade A and clade B. Samples are projected onto the first and second principal components: PC1 (86%) and PC2 (11%). Each group is represented by its perimeter.

In the factor map, two distinct areas can be observed: males are located on the left and females on the right, which is consistent with the fact that females of these insects exhibit larger morphological characteristics than males. Specifically, the females of clade B are the largest of all the specimens studied.

Each community possesses its own morphological identity, supported by the fact that the areas are well-defined and there is a lack of large overlapping regions.

### Molecular and Phylogenetic Analysis

For molecular analysis, partial sequences of the *cox*1, *co*b, and 12S mtDNA genes were amplified and sequenced from 27 individuals. All nucleotide sequence data generated in this study have been deposited in the GenBank, EMBL, and DDBJ databases (Supplementary file 2: Table S2). In addition, the alignments were performed using the sequences obtained in the present study, along with those retrieved from GenBank and the outgroups employed.

The multiple alignment of 80 12S nucleotide sequences resulted in a dataset comprising 566 characters. For *cox*1, 84 taxa were included in the analysis, resulting in a dataset comprising 286 characters. Furthermore, for *co*b, 132 taxa were analyzed, yielding a dataset of 272 characters.

Phylogenetic analyses were performed individually for each marker analyzed (data not shown) and by concatenating these markers (Fig. 3). All trees revealed six clades, or distinct groups, labeled from A to F. After analyzing our obtained sequences, they grouped into two different clades: one in clade A and the other in clade B; however, most of the obtained sequences grouped into clade B. Similarly, clade A was more closely related to clade D than to the other clades. However, clade B was more closely related to clade F (Fig. 3). In the concatenated tree, not all phylogenetic relationships were strongly supported, although they were well supported in the individual trees (data not shown).

**Fig. 3.**
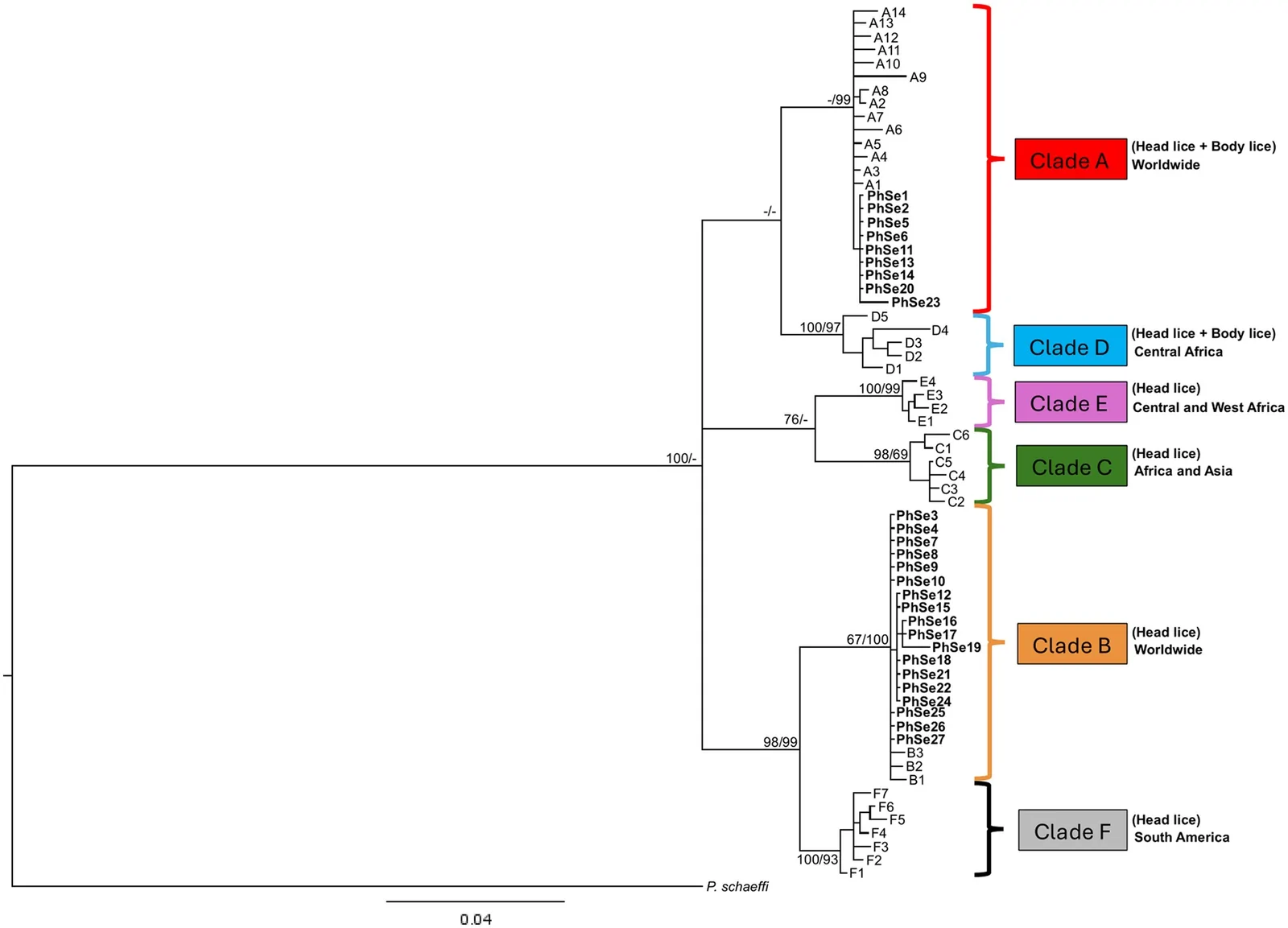
Phylogenetic tree of *Pediculus* species based on combined analysis of 12S, *cox*1, and *co*b inferred using Bayesian Inference. Maximum Likelihood bootstrap values of clades are listed first, followed by Bayesian Posterior Probabilities, respectively, for clade frequencies exceeding 60%.

Analysis of the similarity among 12S sequences, excluding the outgroup, revealed the lowest similarity between *P. humanus* groups C and F, as well as between groups C and D (92.05%). Of the sequences obtained in the present study, the lowest similarity was found with those of *P. humanus* group C (92.23%), while the highest similarity was observed with the sequences from *P. humanus* groups A and B (100%) (Supplementary file 3: Table S3).

Analyzing the similarity among *cox*1 sequences, excluding the outgroup, the lowest similarity was observed between *P. humanus* group A and *P. humanus* group B (90.21%). Furthermore, when comparing our sequences with the rest of the analyzed sequences, the lowest similarity was also with *P. humanus* group A (90.56%). However, the highest similarity was also observed with sequences from groups A and B (100%) (Supplementary file 4: Table S4).

Upon analyzing the similarity among *co*b sequences, excluding the outgroup, the least similarity was detected between *P. humanus* groups B and E, as well as between groups E and F (86.76%). Upon comparing our sequences with the other analyzed sequences, the lowest similarity was also observed with *P. humanus* group E (87.50%). Additionally, the highest similarity was found with groups A and B (100%) (Supplementary file 5: Table S5).

### Resistance Detection

PCRs were able to amplify the kdr partial fragments of 27 head louse samples. All 27 lice analyzed obtained the same nucleotide sequence and were positive for the three different mutation sites found within the analyzed gene (Fig. 4). The nucleotide sequence data generated in this study have been deposited in the GenBank, EMBL, and DDBJ databases (PV784700).

**Fig. 4.**
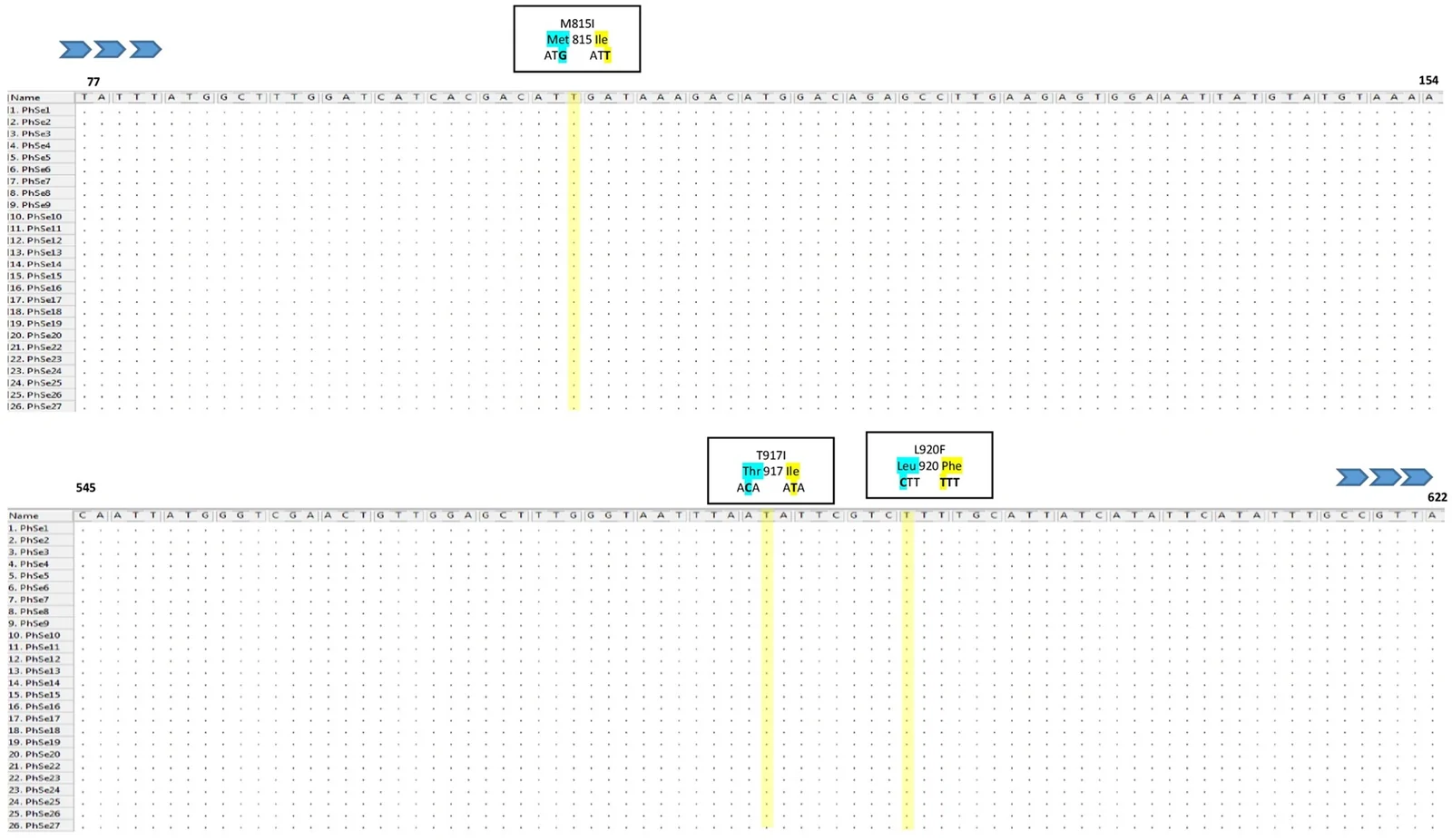
Alignment of partial sequences of the domain fragment of the voltage-sensitive sodium channel (VSSC) α-subunit gene from the head louse. Mutation sites are marked in yellow, and the original unmutated base is in turquoise.

In the present molecular analysis, we identified three key mutations in the VSSC gene of 27 adult specimens of *P. humanus* that are associated with pyrethroid resistance. The M815I mutation involves the substitution of methionine (M) with isoleucine (I) at position 815. The T917I mutation, which substitutes threonine (T) with isoleucine (I) at position 917. Lastly, the L920F mutation involves the substitution of leucine (L) with phenylalanine (F) at position 920. These mutations were found in 100% of the specimens, indicating a widespread resistance mechanism in the studied population.

## Discussion

Head lice infestation remains a common parasitic condition worldwide, affecting people of all ages and across diverse geographic regions. Despite the availability of several treatment options, effective management remains challenging because of factors such as insecticide resistance, treatment failures, and reinfestation.

In this study, we conducted an analysis of a head lice population from Seville, Spain, a region where no genetic or permethrin resistance data had been previously reported.

To assess these phylogenetic relationships, we targeted three mitochondrial markers: *cox*1, *co*b, and 12S rRNA. Mitochondrial markers are widely used for insect phylogeography and clade assignment because their generally rapid evolution, maternal inheritance, and small effective population size can reveal evolutionary lineages and historical population structure (Dong et al. 2021). However, mtDNA should not be treated as universally nonrecombining in *Pediculus*: its mitochondrial genome is fragmented into 18 minicircular chromosomes, and recombination between minicircles has been documented (Shao et al. 2009, Shao and Barker 2011). These loci nevertheless provide an established framework for global mitochondrial-clade identification in *P. humanus* (Light et al. 2008a, Amanzougaghene et al. 2019).

In our analyses, individual-gene trees resolved the six primary *P. humanus* mitochondrial clades (A–F) with congruent overall topologies. Support for intermediate nodes was lower in single-gene trees, plausibly reflecting the limited number of parsimony-informative sites in the shorter *cox*1 (286 bp) and *co*b (272 bp) alignments. The longer 12S alignment (566 bp) showed greater topological stability, whereas *cox*1 and *co*b captured greater inter-clade nucleotide divergence, reaching 9.79% and 13.24%, respectively.

Concatenating the three markers (Fig. 3) increased phylogenetic signal and improved bootstrap and posterior-probability support across the major lineages. This pattern is consistent with comparative *Pediculus* work showing that more extensive mitochondrial sequence data are markedly more informative than short partial-gene sequences, although partial *cox*1 and *co*b fragments can still assign lice to major clades (Xiong et al. 2014). Pairwise identity analyses further supported the genetic structure of the Seville population: sequences assigned to clades A and B showed 100% identity to their respective references, while comparisons with more distant clades, including E and F, reached 13.24% divergence. Together, these results show that the marker set retained sufficient signal to discriminate the mitochondrial lineages represented in this dataset.

According to the results obtained, human head lice are classified into six mitochondrial clades (A–F), each showing a characteristic geographical distribution (Light et al. 2008a, Boyd and Reed 2012, Ashfaq et al. 2015, Amanzougaghene et al. 2019, Hammoud et al. 2022). In the present study, phylogenetic analysis based on mitochondrial markers identified two clades, A and B, among the lice collected, with clade B being the predominant lineage. This pattern is consistent with previous studies from Europe, including France, where both clades A and B have also been reported, although clade A was more prevalent in those populations (Ashfaq et al. 2015, Amanzougaghene et al. 2016, 2019, Candy et al. 2018).

Only one host presented a mixed infestation, with one female louse belonging to clade A and one male louse belonging to clade B. In all remaining hosts, all analyzed lice belonged exclusively to a single mitochondrial clade. This isolated mixed infestation is consistent with co-infestation following exposure to more than one transmission source where clades A and B occur sympatrically. It also places lice from the two clades on the same host and therefore creates an opportunity for inter-clade mating. Molecular population-genetic studies have reported clades A and B in sympatry and nuclear-marker patterns consistent with recombination, supporting the inference that mitochondrial clades do not constitute reproductively isolated lineages (Boutellis et al. 2015). Similar evidence for recombination between sympatric mitochondrial clades has also been reported for clades A and C (Veracx et al. 2013). Because mtDNA is maternally inherited, progeny of an A-clade female would retain her mitochondrial lineage even if nuclear genes were inherited from both parents. In addition, no lice belonging to clade F were detected in the analyzed samples. However, the demographic background of the participants was not recorded; therefore, no conclusions can be drawn regarding the absence of this clade in the study population. On the other hand, morphological differences between lice are subtle and typically discernible only through discriminant function analysis (Reed et al. 2004), with a few notable traits such as body length and the length of the tibia of the middle legs aiding in distinguishing head lice from body lice (Busvine 1978).

In the present study, geometric morphometrics successfully differentiated *P. humanus* specimens belonging to clades A and B. However, these findings should be interpreted with caution because of the limited number of adult specimens, particularly from clade A, which may not fully represent the morphological variability within each clade. In addition, it cannot be ruled out that factors such as insecticide resistance may have contributed to the observed morphological variation. Future studies including larger and more balanced sample sizes are needed to confirm these findings and to further investigate the relationship between mitochondrial lineage, insecticide resistance, and morphology.

The application of insecticides remains a key strategy for managing and preventing louse infestations. However, frequent use of these chemicals has contributed to the development of resistance (Fu et al. 2022). As a result, the effectiveness of many insecticides has diminished due to the rise of resistant lice populations, which has, in part, led to an increase in head louse infestations across various regions (Durand et al. 2012). Although permethrin continues to be widely used in some countries, including Spain, first-line treatment recommendations for pediculosis capitis vary internationally according to national guidelines and local resistance patterns (Brownell et al. 2020). Lice develop resistance through multiple mechanisms, including kdr to permethrin and malathion resistance via elevated carboxylesterase activity (Durand et al. 2012, Kwon et al. 2014). The resistance of head lice to pyrethroids is primarily linked to kdr and is associated with three-point mutations (M815I, T917I, and L920F) in the VSSC α-subunit (Roca-Acevedo et al. 2019, Larkin et al. 2020). The M815I mutation is in the IIS1-2 extracellular loop of the VSSC and contributes to reduced sensitivity to pyrethroids; the T917I mutation is a well-known kdr mutation that significantly decreases the efficacy of pyrethroids by altering the target site. Lastly, the L920F mutation further contributes to resistance by modifying the sodium channel’s structure (Firooziyan et al. 2017).

In our study, a partial sequence of the VSSC α-subunit gene was employed to screen for mutations associated with permethrin resistance in a population of 27 head lice. All analyzed specimens carried the three mutations previously linked to pyrethroid resistance. These findings provide evidence for the occurrence of resistance-associated alleles in head lice collected in Seville and suggest that selective pressure exerted by pyrethroid-based treatments may have contributed to their persistence in this population. However, given the limited sample size, these results should not be interpreted as an estimate of mutation frequency in the wider head lice population.

In comparison, similar patterns of pyrethroid resistance have been reported in other countries. High frequencies of the M815I, T917I and L920F mutations have been detected in head lice populations from Iran and Turkey, while slightly lower frequencies have been reported in Argentina (Karakuş et al. 2020, Ghavami et al. 2023, Toloza et al. 2024). In contrast, Thailand showed a markedly lower prevalence of the T917I mutation (31%) (Brownell et al. 2020). Overall, these findings indicate that kdr mutations are widespread worldwide, although their prevalence varies considerably across regions, highlighting the importance of local surveillance to guide treatment strategies.

Additionally, novel mutations such as K794E, F815I, and N818D, affecting other amino acid positions, have been ­identified (Khaligh et al. 2021). Resistance to ivermectin has also been observed and is linked to three non-synonymous mutations (A251V, H272R and S46P) in the GluCl gene (Amanzougaghene et al. 2018).

## Conclusions

This study provides the first molecular and morphometric characterization of head lice (*P. h. capitis*) from the Seville (Spain) study area. Our phylogenetic analysis identified the presence of two distinct mitochondrial lineages, clades A and B, with clade B being the predominant lineage among the analyzed samples. In addition, kdr resistance-associated alleles (M815I, T917I, and L920F) were detected in all lice analyzed for the VSSC gene, providing evidence of the occurrence of permethrin resistance-associated mutations in head lice from this population. Although these findings are based on a limited number of samples, they highlight the importance of continued surveillance of insecticide resistance in local head lice populations and provide baseline molecular data for future studies.

Finally, the significant morphometric differences found between clades validate the utility of geometric morphometrics as a complementary tool to molecular methods for population studies. These integrated insights are essential for developing more effective, evidence-based control programs to manage head lice infestations and monitor their impact on public health.

## Supplementary Material

ieag110_Supplementary_Data
