# Supplementary material for: Genetic diversity and occurrence of permethrin resistance-associated mutations in a population of head lice (Pediculus humanus capitis) from Seville, Spain
Source: J Insect Sci. 2026 Oct 1;26(5):ieag110. doi: 10.1093/jisesa/ieag110 (PMC13628266; doi:10.1093/jisesa/ieag110)
Supplement: Supplementary file 1 [file ieag110_supplementary_data.zip › Supp. Table S1.docx]

**Table S1. *Pediculus humanus* sequences obtained in this study and from the GenBank database used for molecular and phylogenetic analysis.**

| ID sample (this study) | ID GenBank | 12S | *cox*1 | *co*b |
| --- | --- | --- | --- | --- |
| PhSe1 | Ph2 | PQ575774 | PQ560604 | PQ568347 |
| PhSe2 | Ph3 | PQ575775 | PQ560605 | PQ568348 |
| PhSe3 | Ph28 | PQ575776 | PQ560606 | PQ568349 |
| PhSe4 | Ph31 | PQ575777 | PQ560607 | PQ568350 |
| PhSe5 | Ph38 | PQ575778 | PQ560608 | PQ568351 |
| PhSe6 | Ph39 | PQ575779 | PQ560609 | PQ568352 |
| PhSe7 | Ph45 | PQ575780 | PQ560610 | PQ568353 |
| PhSe8 | Ph50 | PQ575781 | PQ560611 | PQ568354 |
| PhSe9 | Ph51 | PQ575782 | PQ560612 | PQ568355 |
| PhSe10 | Ph60 | PQ575783 | PQ560613 | PQ568356 |
| PhSe11 | Ph71 | PQ575784 | PQ560614 | PQ568357 |
| PhSe12 | Ph111 | PQ575785 | PQ560615 | PQ568358 |
| PhSe13 | Ph244 | PQ575786 | PQ560616 | PQ568359 |
| PhSe14 | Ph283 | PQ575787 | PQ560617 | PQ568360 |
| PhSe15 | Ph324 | PQ575788 | PQ560618 | PQ568361 |
| PhSe16 | Ph331 | PQ575789 | PQ560619 | PQ568362 |
| PhSe17 | Ph363 | PQ575790 | PQ560620 | PQ568363 |
| PhSe18 | Ph477 | PQ575791 | PQ560621 | PQ568364 |
| PhSe19 | Ph65 | PQ575792 | PQ560622 | PQ568365 |
| PhSe20 | Ph80 | PQ575793 | PQ560623 | PQ568366 |
| PhSe21 | Ph135 | PQ575794 | PQ560624 | PQ568367 |
| PhSe22 | Ph249 | PQ575795 | PQ560625 | PQ568368 |
| PhSe23 | Ph287 | PQ575796 | PQ560626 | PQ568369 |
| PhSe24 | Ph303 | PQ575797 | PQ560627 | PQ568370 |
| PhSe25 | Ph329 | PQ575798 | PQ560628 | PQ568371 |
| PhSe26 | Ph364 | PQ575799 | PQ560629 | PQ568372 |
| PhSe27 | Ph481 | PQ575800 | PQ560630 | PQ568373 |
| A1 | *Pediculus humanus* | MH428970 | KM579493 | KM579538 |
| A2 | *Pediculus humanus* | MH428971 | KM579500 | KM579539 |
| A3 | *Pediculus humanus* | MH428972 | KM579501 | KM579540 |
| A4 | *Pediculus humanus* | MH428973 | KM579502 | KM579541 |
| A5 | *Pediculus humanus* | MH428974 | KM579503 | KM579542 |
| A6 | *Pediculus humanus* | MH428975 | KM579504 | KM579543 |
| A7 | *Pediculus humanus* | MH428976 | KM579505 | KM579544 |
| A8 | *Pediculus humanus* | MH428977 | KM579506 | KM579545 |
| A9 | *Pediculus humanus* | MH428978 | MH429022 | KM579553 |
| A10 | *Pediculus humanus* | MH428980 | KM579507 | KM579555 |
| A11 | *Pediculus humanus* | MH428981 | KM579494 | KM579547 |
| A12 | *Pediculus humanus* | MH428982 | KM579495 | KM579548 |
| A13 | *Pediculus humanus* | MH428983 | KM579496 | KM579549 |
| A14 | *Pediculus humanus* | MH428984 | KM579497 | KM579550 |
| B1 | *Pediculus humanus* | MH428993 | KM579509 | KX249763 |
| B2 | *Pediculus humanus* | MH428994 | KM579508 | KM579556 |
| B3 | *Pediculus humanus* | MH428995 | KM579511 | KM579557 |
| C1 | *Pediculus humanus* | MH429004 | KM579523 | KM579561 |
| C2 | *Pediculus humanus* | MH429005 | KM579524 | KM579562 |
| C3 | *Pediculus humanus* | MH429006 | MG759557 | KM579563 |
| C4 | *Pediculus humanus* | MH429007 | MG759558 | KM579564 |
| C5 | *Pediculus humanus* | MH429008 | MH429033 | KM579565 |
| C6 | *Pediculus humanus* | MH429009 | MH429034 | KX249778 |
| D1 | *Pediculus humanus* | MH428985 | MG759562 | KX249766 |
| D2 | *Pediculus humanus* | MH428986 | MH429025 | KX249767 |
| D3 | *Pediculus humanus* | MH428987 | MG759561 | KX249768 |
| D4 | *Pediculus humanus* | MH428989 | MH429024 | KX249769 |
| D5 | *Pediculus humanus* | MH428990 | MH429023 | KX249770 |
| E1 | *Pediculus humanus* | MH429014 | MG759559 | KX249780 |
| E2 | *Pediculus humanus* | MH429015 | MG759560 | KX249781 |
| E3 | *Pediculus humanus* | MH429016 | MH429035 | KM579560 |
| E4 | *Pediculus humanus* | MH429017 | MG759563 | KY937987 |
| F1 | *Pediculus humanus* | MH428996 | MH429026 | MH429040 |
| F2 | *Pediculus humanus* | MH428997 | MH429027 | MH429041 |
| F3 | *Pediculus humanus* | MH428998 | MH429028 | MH429042 |
| F4 | *Pediculus humanus* | MH428999 | MH429029 | MH429043 |
| F5 | *Pediculus humanus* | MH429000 | MH429030 | MH429044 |
| F6 | *Pediculus humanus* | MH429001 | MH429031 | MH429045 |
| F7 | *Pediculus humanus* | MH429002 | MH429032 | MH429046 |
| *Pediculus schaeffi* | *Pediculus schaeffi* | KR706169 | AY695999 | FJ267434 |
