# Supplementary material for: Genetic diversity and occurrence of permethrin resistance-associated mutations in a population of head lice (Pediculus humanus capitis) from Seville, Spain
Source: J Insect Sci. 2026 Oct 1;26(5):ieag110. doi: 10.1093/jisesa/ieag110 (PMC13628266; doi:10.1093/jisesa/ieag110)
Supplement: Supplementary file 1 [file ieag110_supplementary_data.zip › Supp. Table S2.docx]

**Table S2.** Summary of ID sample, sequence lengths and accession numbers obtained in this study.

| **ID sample** | **Sequence length (bp)** | | | | | | **Accession number** | | | | | |
| --- | --- | --- | --- | --- | --- | --- | --- | --- | --- | --- | --- | --- |
|  | **12S** | | ***cox*1** | | ***co*b** | | **12S** | | ***cox*1** | | ***co*b** | |
| PhSe1 | 557 | 788 | | 305 | | PQ575774 | | PQ560604 | | PQ568347 | |  |
| PhSe2 | 557 | 788 | | 305 | | PQ575775 | | PQ560605 | | PQ568348 | |  |
| PhSe3 | 557 | 788 | | 305 | | PQ575776 | | PQ560606 | | PQ568349 | |  |
| PhSe4 | 557 | 788 | | 305 | | PQ575777 | | PQ560607 | | PQ568350 | |  |
| PhSe5 | 557 | 788 | | 305 | | PQ575778 | | PQ560608 | | PQ568351 | |  |
| PhSe6 | 557 | 788 | | 305 | | PQ575779 | | PQ560609 | | PQ568352 | |  |
| PhSe7 | 557 | 788 | | 305 | | PQ575780 | | PQ560610 | | PQ568353 | |  |
| PhSe8 | 557 | 788 | | 305 | | PQ575781 | | PQ560611 | | PQ568354 | |  |
| PhSe9 | 557 | 788 | | 305 | | PQ575782 | | PQ560612 | | PQ568355 | |  |
| PhSe10 | 557 | 788 | | 305 | | PQ575783 | | PQ560613 | | PQ568356 | |  |
| PhSe11 | 557 | 788 | | 305 | | PQ575784 | | PQ560614 | | PQ568357 | |  |
| PhSe12 | 557 | 788 | | 305 | | PQ575785 | | PQ560615 | | PQ568358 | |  |
| PhSe13 | 557 | 788 | | 305 | | PQ575786 | | PQ560616 | | PQ568359 | |  |
| PhSe14 | 557 | 788 | | 305 | | PQ575787 | | PQ560617 | | PQ568360 | |  |
| PhSe15 | 557 | 788 | | 305 | | PQ575788 | | PQ560618 | | PQ568361 | |  |
| PhSe16 | 556 | 788 | | 305 | | PQ575789 | | PQ560619 | | PQ568362 | |  |
| PhSe17 | 557 | 788 | | 305 | | PQ575790 | | PQ560620 | | PQ568363 | |  |
| PhSe18 | 557 | 788 | | 305 | | PQ575791 | | PQ560621 | | PQ568364 | |  |
| PhSe19 | 557 | 788 | | 305 | | PQ575792 | | PQ560622 | | PQ568365 | |  |
| PhSe20 | 557 | 788 | | 305 | | PQ575793 | | PQ560623 | | PQ568366 | |  |
| PhSe21 | 557 | 788 | | 305 | | PQ575794 | | PQ560624 | | PQ568367 | |  |
| PhSe22 | 556 | 788 | | 305 | | PQ575795 | | PQ560625 | | PQ568368 | |  |
| PhSe23 | 557 | 788 | | 305 | | PQ575796 | | PQ560626 | | PQ568369 | |  |
| PhSe24 | 556 | 788 | | 305 | | PQ575797 | | PQ560627 | | PQ568370 | |  |
| PhSe25 | 556 | 788 | | 305 | | PQ575798 | | PQ560628 | | PQ568371 | |  |
| PhSe26 | 556 | 788 | | 305 | | PQ575799 | | PQ560629 | | PQ568372 | |  |
| PhSe27 | 557 | 788 | | 305 | | PQ575800 | | PQ560630 | | PQ568373 | |  |
